# Supplementary material for: Identification of photoperiod-induced specific miRNAs in the adrenal glands of Sunite sheep (Ovis aries)
Source: Front Vet Sci. 2022 Jul 22;9:888207. doi: 10.3389/fvets.2022.888207 (PMC9354845; doi:10.3389/fvets.2022.888207)
Supplement: Supplementary file 1 [file Data_Sheet_1.ZIP › Supplementary file/miRDeep2 file.zip!/miRDeep2 file/Know_Structure/SPLP42A2/oar-mir-26b.pdf]

miRBase precursor : oar-mir-26b
Total read count : 190181
oar-miR-26b read count : 189997
remaining reads : 184

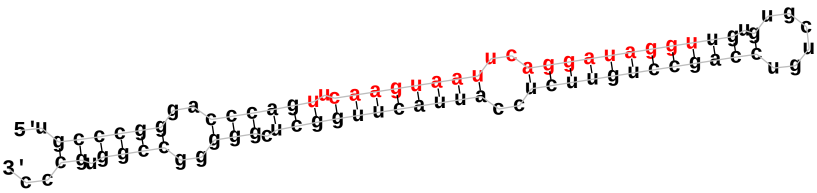

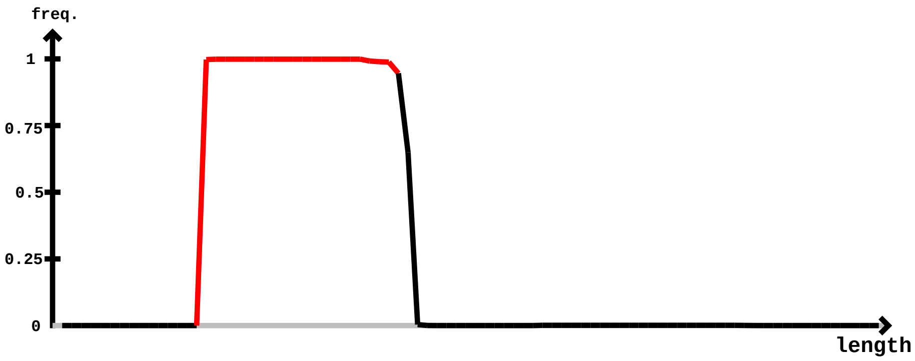

oar-miR-26b

```
5'- ugcccgggacccaguucaaguaauucaggauagguugugugcuguccagccuguucuccauuacuuggcucgggggccggugccc   -3'    exp
    .((((((..((((((.((((((((..(((((((((((.(......))))))))))))..))))))))))).))).)))).))..    reads     mm     sample
    .............guucaaguaauCcaggauagg..................................................    1         1      seq
    ..............uucaaguaauucaggUu.....................................................    1         1      seq
    ..............uucaaguaauucaggaA.....................................................    17        1      seq
    ..............uucaaguaauucaggCu.....................................................    1         1      seq
    ..............uucaaguGauucaggau.....................................................    1         1      seq
    ..............Aucaaguaauucaggau.....................................................    4         1      seq
    ..............uucaaguaauucaggaC.....................................................    129       1      seq
    ..............uucaagCaauucaggau.....................................................    1         1      seq
    ..............uucaaguaUuucaggau.....................................................    2         1      seq
    ..............uucaaguaauuUaggau.....................................................    1         1      seq
    ..............uucaagAaauucaggau.....................................................    1         1      seq
    ..............uucaaguaauucagUau.....................................................    1         1      seq
    ..............uucaGguaauucaggau.....................................................    4         1      seq
    ..............uCcaaguaauucaggau.....................................................    2         1      seq
    ..............uucaaguaCuucaggau.....................................................    1         1      seq
    ..............uucaaguaauucaggau.....................................................    1043      0      seq
    ..............uuUaaguaauucaggau.....................................................    5         1      seq
    ..............uucCaguaauucaggau.....................................................    74        1      seq
    ..............uucaaguaauucaggaG.....................................................    54        1      seq
    ..............uucaaguaauucaggaua....................................................    439       0      seq
    ..............uucaaguaauucaggaAa....................................................    3         1      seq
    ..............uucaaguaauucaggaCa....................................................    2         1      seq
    ..............uucCaguaauucaggaua....................................................    27        1      seq
    ..............uucaaguaauucaggauag...................................................    279       0      seq
    ..............uucaaguaauucaggauagg..................................................    7979      0      seq
    ..............uucaaguaauucaggauaggu.................................................    56241     0      seq
    ..............uucaaguaauucaggauagguu................................................    122666    0      seq
    ..............uucaaguaauucaggauagguug...............................................    671       0      seq
    ..............uucaaguaauucaggauagguugu..............................................    47        0      seq
    ...............ucaaguaauucaggauagg..................................................    10        0      seq
    ...............ucaaguaauucaggauaggu.................................................    63        0      seq
    ...............ucaaguaauucaggauagguu................................................    170       0      seq
    ...............ucaaguaauucaggauagguug...............................................    7         0      seq
    ...............ucaaguaauucaggauagguugu..............................................    1         0      seq
    ................caaguaauucaggauagg..................................................    2         0      seq
```

oar-miR-26b

```
ugcccgggacccaguucaaguaauucaggauagguugugugcuguccagccuguucuccauuacuuggcucgggggccggugccc
................caaguaauucaggauaggu..................................................   11   0   seq
................caaguaauucaggauagguu.................................................   21   0   seq
.................aaguaauucaggauaggu..................................................   1    0   seq
.................aaguaauucaggauagguu.................................................   14   0   seq
.....................................ugugcuguccagccuguucuccauuacuuggcu...............   1    0   seq
...............................................agccuguucuccauuacuug..................   1    0   seq
.................................................ccuguucuccauuacuuggc................   12   0   seq
.................................................ccuguucuccauuacuuggcu...............   73   0   seq
.................................................ccuguucuccauuacuuggcuc..............   74   0   seq
.................................................ccuguucuccauuacuuggcucg.............   23   0   seq
```
